# Human rhinovirus spatial-temporal epidemiology in rural coastal Kenya, 2015-2016, observed through outpatient surveillance

**DOI:** 10.12688/wellcomeopenres.14836.2

**Published:** 2019-03-27

**Authors:** John Mwita Morobe, Joyce U. Nyiro, Samuel Brand, Everlyn Kamau, Elijah Gicheru, Fredrick Eyase, Grieven P. Otieno, Patrick K. Munywoki, C.N. Agoti, D.J. Nokes

**Affiliations:** 1 Institute of Biotechnology Research, Jomo Kenyatta University of Agriculture and Technology, Juja, +254, Kenya; 2 Epidemiology and Demography Department, KEMRI-Wellcome Trust Research Programme, Kilifi, +254, Kenya; 3 Zeeman Institute of Systems Biology and Infectious Disease Research (SBIDER), University of Warwick, Coventry, UK; 4 School of Life Sciences, University of Warwick, Coventry, UK; 5 Public Health, Pwani University, Kilifi, +254, Kenya

**Keywords:** Human rhinovirus, Out-patient, Acute respiratory infection, Surveillance, Spatial patterns, Coastal Kenya

## Abstract

**Background: **Human rhinovirus (HRV) is the predominant cause of upper respiratory tract infections, resulting in a significant public health burden. The virus circulates as many different types (168), each generating strong homologous, but weak heterotypic, immunity. The influence of these features on transmission patterns of HRV in the community is understudied.

**Methods: **Nasopharyngeal swabs were collected from patients with symptoms of acute respiratory infection (ARI) at nine out-patient facilities across a Health and Demographic Surveillance System between December 2015 and November 2016. HRV was diagnosed by real-time RT-PCR, and the VP4/VP2 genomic region of the positive samples sequenced. Phylogenetic analysis was used to determine the HRV types. Classification models and G-test statistic were used to investigate HRV type spatial distribution. Demographic characteristics and clinical features of ARI were also compared.

**Results: **Of 5,744 NPS samples collected, HRV was detected in 1057 (18.4%), of which 817 (77.3%) were successfully sequenced. HRV species A, B and C were identified in 360 (44.1%), 67 (8.2%) and 390 (47.7%) samples, respectively. In total, 87 types were determined: 39, 10 and 38 occurred within species A, B and C, respectively. HRV types presented heterogeneous temporal patterns of persistence. Spatially, identical types occurred over a wide distance at similar times, but there was statistically significant evidence for clustering of types between health facilities in close proximity or linked by major road networks.

**Conclusion: **This study records a high prevalence of HRV in out-patient presentations exhibiting high type diversity. Patterns of occurrence suggest frequent and independent community invasion of different types. Temporal differences of persistence between types may reflect variation in type-specific population immunity. Spatial patterns suggest either rapid spread or multiple invasions of the same type, but evidence of similar types amongst close health facilities, or along road systems, indicate type partitioning structured by local spread.

## Introduction

Human rhinovirus (HRV) is the predominant cause of upper respiratory tract infections (URTIs) referred to as the common cold ^1– 4^. The virus has also been associated with lower respiratory tract illnesses, including exacerbation of asthma and chronic obstructive pulmonary disease ^5, 6^. HRVs have also been identified in mild and asymptomatic cases ^7^. HRV is globally ubiquitous and infections usually occur all year-round, although peaking in the early autumn and late spring in many temperate or subtropical countries, and in the rainy season in tropical countries ^8– 10^. Despite most HRV infections being mild, they pose a considerable social and economic burden due to time lost from work or school, medical attendance and reduced performance of regular duties ^11^. HRV transmission, infection patterns and diversity have been rarely studied in low-income settings despite bearing the majority burden of acute respiratory illnesses (ARI).

HRVs fall under the genus *Enterovirus* (HEVs) in the family *Picornaviridae* ^12^. Their genome occurs as a positive-sense, single-stranded RNA molecule of approximately 7.2 kb flanked by a 5’ untranslated region (UTR) and 3’ poly-A tail. There are three HRV species denoted as HRV-A, HRV-B and HRV-C within which over 168 genetically distinct types have been identified ^13^. Identification of these types presently relies on molecular typing methods based on sequence analysis of VP1 or VP4/VP2 proteins encoding regions ^12, 14^.

Studies investigating the transmission of HRV show that multiple infections frequently occur in individuals over short periods of time of the order of a few weeks ^15– 17^. In household studies, family members experience rapid rates of reinfection (for instance, up to 5 infections in adults or 12 infections in young children per year) where each infection tends to be caused by a different HRV-type and rarely with the same HRV-type ^4, 18– 20^. At a population level of observation, repeated reinfections in individuals appears to reflect the introduction and circulation of new types in the community, rather than persistence of the same HRV types ^21– 24^. These observations are consistent with evidence from immunological studies on HRV that indicate strong homologous type responses and little heterotypic immunity to protect individuals against multiple types ^25– 27^. Moreover, studies have shown that individuals develop relatively long-lasting (~ 1 year) type-specific neutralizing antibodies (IgG and IgA) against a specific infecting HRV-type but susceptibility to other different HRV types remains and infection will occur as so long as there is an exposure ^28, 29^.

Hence it might be hypothesized that at the population level the virus types spread in a manner independent of each other. However, the statistical evidence to support this assertion has yet to be undertaken. This would require spatially structured study designs to capture temporal-spatial HRV transmission. Data of this sort could improve understanding on the nature of spread of these viruses at the population level in relation to homotypic and heterotypic immunity.

A recently published study ^30^, described the epidemiology of respiratory viruses in coastal Kenya through surveillance of acute respiratory infection presentations at outpatient facilities spatially structured across the well-defined population of the Kilifi Health and Demographic Surveillance System (KHDSS) ^31^. The present study utilized HRV positive samples from this surveillance between December 2015 through November 2016, to explore the temporal and spatial circulation patterns and genetic diversity of HRV types in the wider Kilifi community.

## Methods

### Study area

The study was undertaken within Kilifi County in Coastal Kenya, at nine health facilities namely: Matsangoni, Ngerenya, Mtondia, Sokoke, Mavueni, Jaribuni, Chasimba, Pingilikani and Junju, all located within the KHDSS as previously described ^30^. The county has a population of about 1,109,735 and is predominantly rural; the main economic activities include fishing, tourism and subsistence farming. The area has a tropical climate with two rainy seasons (long rains in April to July and short rains in October to December). The KHDSS area in which the study was restricted covers 891km ^2^ and has a population of 287,014 (mid-point 2016) which is heterogeneously distributed with the highest density in Kilifi township ^30^.

### Study design

Nasopharyngeal swab (NPS) samples and demographic data were collected from patients presenting with ARI symptoms at the nine selected health facilities within the KHDSS between December 2015 and November 2016 inclusive. The KHDSS area has 21 public health facilities operating under the Kenya Ministry of Health (MOH); the 9 health facilities were purposively selected to participate in this study to give a broad representation across the geographical region and considering key road networks and population densities as described in the previous publication ^30^. Recruitment of study participants occurred during the routine clinical reviewing procedure by the resident clinician or nurse at each health facility. Informed consent was sought from participants or their caregiver (if the patient was less than 18 years of age) and who met the inclusion criteria. Patients were eligible for inclusion in the study if presenting with one or more of the following symptoms: cough, sneezing, nasal congestion, difficulty in breathing, or fast breathing for age as defined by World Health Organisation (WHO) guidelines ^32^. Individuals were excluded if aged less than 7 days old or if the ARI symptoms had been observed for more than 30 days.

A maximum of 15 participants were recruited per facility per week. Demographic and clinical history details of the participants were collected from eligible patients as described previously ^30^. A NPS sample was collected by inserting a sterile nylon flocked plastic-shafted swab (503CS01, Copan Diagnostics, Flocked Swab Technologies, Italy) into one nostril to a distance where the tip located the deep nasopharynx and twisting 3 times before gently removing (taking 5–10 seconds). The NPS sample was stored in universal virus transport media and kept at approximately 8°C in an ice packed cool box. It was then delivered to the KEMRI–Wellcome Trust Research Programme laboratories approximately 4 hours after collection and stored at −80°C.

### Ethical considerations

The study protocol was approved by the KEMRI-Scientific and Ethics Review Unit (SERU# 3103) and the University of Warwick Biomedical and Scientific Research Ethics Committee (BSREC# REGO-2015-6102). Written informed consent was gathered from all patients or their caregiver.

### Laboratory procedures

Viral RNA was extracted from each sample using QIAamp Viral RNA kit (Qiagen Inc., Valencia, California, USA, Catalog number: 52906) according to the manufacturer’s instructions. Diagnostic PCR was performed to confirm the presence of HRV genetic material using real-time RT-PCR with the following primers and probes; forward primer (TGGACAGGGTGTGAAGAGC, reverse primer (CAAAGTAGTCGGTCCCATCC) and probe (VIC-TCCTCCGGCCCCTGAATG-TAMRA) (Applied Biosystems, United Kingdom, Catalog number 00-45-0818, 00-45-0819 and 00-45-0820) targeting the 5’ UTR using the following thermocycling conditions; 20 min at 50°C for reverse transcription, 5 min for 95°C for polymerase activation and 40 cycles of 15s at 95°C and 30s at 60°C as previously described ^33, 34^. The PCR runs included positive control (of known DNA copies) of the targeted 5’UTR product generated in-house. Samples were quantified based on comparison to the positive control sample. Samples were considered positive if Ct value <= 35.0 ^30, 34^. To amplify VP4/VP2 coding region was amplified for the HRV-positive samples, we used a single step PCR adapted from a nested VP4/VP2 -PCR assay previously described ^35^. Forward primer OS (CCGGCCCCTGAATGYGGCTAA) and reverse primer IAS (TCWGGHARYTTCCAMCACCANCC) (Invitrogen, United Kingdom, catalog number 00-45-0816 and 00-45-0817) were used in a RT-PCR reaction using Qiagen OneStep RT-PCR kit (Qiagen Valencia, Catalog number: 210212). Thermocycling conditions were set as follows: Reverse transcription of the RNA to cDNA at 50°C for 30 minutes, in-activation of reverse transcriptase and activation of Taq polymerase at 95°C for 15 minutes. This was followed by 40 cycles of denaturation at 94°C for 1 minute, annealing at 60°C for 1 minute and strand extension at 72°C for 1 min and final incubation/extension at 72°C for 10 minutes. Purified PCR products were sequenced using Big Dye Terminator 3.1 chemistry (Applied Biosystems, Foster City, California, USA, Catalog number 4337455) using the PCR primers in both forward and reverse direction in an ABI Prism 3130×l Genetic Analyzer (Applied Biosystems, Foster City, California, USA).

### Sequence alignment and phylogenetic analysis

Raw sequence reads were assembled into contigs using Sequencher software (version 5, Gene Codes Corporation, Ann Arbor, USA). Multiple sequence alignments (MSA) were prepared using MAFFT v7.220 ^36^. Phylogenetic trees were constructed in MEGA v.6.0 ^37^ with maximum likelihood methods under the GTR model and branch support was assessed using 1000 bootstrap iterations. Types were assigned based on >90% nucleotide similarity to rhinovirus prototype sequences (also referred to as reference sequences) ^13, 14^ and phylogenetic clustering with the reference sequences (with a bootstrap support value above 70%).

### Statistical analysis

Initial statistical analysis was conducted in STATA version 13.1 (College Station, Texas). Statistical comparison of demographic characteristics and clinical features in HRV species was conducted using chi square test of association and Fisher’s exact test where applicable. Frequency distribution and temporal pattern graphs for HRV were generated in R 3.5.1.

### Spatial distribution analysis

Analysis centered on investigating spatial heterogeneity of HRV detections of HRV types, between health facilities. Our null hypothesis was that HRV is introduced and spreads randomly within a population, and that HRV types circulate independent of each other. Hence, we asked: a) Is there evidence that any health facility had significantly more HRV detections in the sample of NPSs than others? b) Are the frequencies of HRV types found per health facility noticeably different from independent random mixing? c) Moreover, was the pattern of types detected at each facility similar to nearby health facilities compared to further away health facilities? To answer these questions we used a combination of the classification algorithms provided by the Orange data mining toolbox ^38^ and logistic regression implemented by the MATLAB v9.4 **fitglme** function. The null hypothesis of independent random HRV type detection was tested using the G-test statistic associated with the contingency table of type frequency per health facility. Type pattern similarity between health facilities was analysed using multi-dimensional scaling (MDS) implement in Orange v3.0. MDS represents the similarity between high-dimensional data-points optimally on a low dimensional (in this case two dimensional) Euclidean metric space, thereby revealing patterns and hidden dimensions in the data ^39^. The Orange implementation of MDS includes a pairwise similarity network linking all points whose high-dimensional similarity is close compared to the typical similarity between any two data-points; this is a threshold quantity set to the algorithm’s maximum therefore including all pairwise connections inferred by the Orange MDS algorithm in our analysis.

### Nucleotide sequence data set

All newly obtained sequences in this study have been deposited into GenBank under accession numbers MH459421-MH460237.

## Results

Between December 2015 and November 2016 inclusive, a total of 5750 participants were recruited and NPS samples collected from 5744 (99.9%) participants as shown in Table 1. Of the 5744 samples collected, 5741 (99.9%) were tested for HRV. HRV was detected in 1057 (18.5%) of the samples tested. Of the 1057, 817 (77.3%) were successfully amplified and sequenced for the VP4/VP2 genomic region; the remaining 240 (22.7%) samples either totally failed amplification or had short consensus sequences recovered (<300 nucleotides) and were not included in phylogenetic analysis.

**Table 1.** Numbers per month; numbers of participants recruited in the study, samples collected from the recruited participants, sample tested and sample identified as human rhinovirus (HRV) positive per month.

Month	Total number of participants recruited	Total number of samples collected	Total number of samples tested	Total number of sample identified as HRV positive
**December**	153	153	153	11
**January**	335	333	332	41
**February**	466	465	465	89
**March**	457	457	457	72
**April**	446	445	445	87
**May**	468	468	467	86
**June**	557	555	555	66
**July**	542	542	541	70
**August**	607	607	607	144
**September**	579	579	579	164
**October**	566	566	566	138
**November**	574	574	574	89
**Total**	**5750**	**5744**	**5741**	**1057**

Among the HRV positive cases, the median age was 2.0 years, 64.0% were under 5 years of age, and 53.9% were females ( Table 2). The percentage of samples found to be of species A, B and C was 44.1 % ( 360), 8.2% (67) and 47.7% (390), respectively, and did not differ by age or sex ( Table 2). Cough (94.5%) and nasal discharge (79.9%) were the most common clinical presentations. Difficulty in breathing, chest in-drawing, crackles, lethargy, nasal flaring, sore throat, wheezing were also recorded ( Table 2). Clinical presentations differed significantly between the typed HRV samples for nasal discharge (p< 0.012) and difficult breathing (p< 0.022) There was no significant (p < 0.05) difference between species A and C (predominant species) for most of the clinical signs reported, except for nasal discharge (p< 0.0042) and difficult breathing (p<0.0194) ( Supplementary Table 1, Supplementary File 1).

**Table 2.** Demographic characteristics of human rhinovirus (HRV) positive and sequenced numbers obtained from patient presenting to nine out-patient health facilities in the Kilifi Health and Demographic Surveillance System, Coastal Kenya between December 2016 and November 2017.

		Total HRV	HRV sequenced				
		n=1057	Total typed n=817	HRV-A n=360	HRV-B n=67	HRV-C n=390	p-value ^3^
**Demographic** **Characteristics**							
**Age (Years)**	**Mean ^1^**	8.3 (14.3)	8.1 (14.3)	9.05(14.91)	8.31(13.50)	7.29 (13.76)	
	**Median ^2^**	2 (0–9)	2 (0–8)	2 (0–13)	2(0–12)	2(0–7)	
**Sex (%)**	**Female**	570 (53.93)	441 ( 53.98)	194 (43.99)	38 (8.62)	209 (47.39)	0.893
	**Male**	487 (46.07)	376 (46.02)	166 (44.15)	29 (7.71)	181 (48.14)	
**Age Bands (%)**	**<1**	317 (29.99)	248 (30.35)	105 (42.34)	24 (9.68)	119 (47.98)	0.192
	**1–4yrs**	359 (33.96)	285 (34.88)	120 (42.11)	19 (6.67)	146 (51.23)	
	**5–9yrs**	125 (11.83)	94 (11.51)	38 (40.43)	6 (6.38)	50 (53.19)	
	**10–15yrs**	57 (5.39)	38 (4.65)	16 (42.11)	3 (7.89)	19 (50)	
	**>15yrs**	199 (18.83)	152 ( 18.6)	81 (53.29)	15 (9.87)	56 (36.84)	
**Notes**	^1^Mean reported with SD in brackets						
	^2^Median reported with IQR in brackets						
	^3^chi square test of association/Fisher's exact test was used test the association between the HRV species, sex and age. No significant associations between species, sex and age were identified ( *P* < 0.05).						

**Figure 1.**
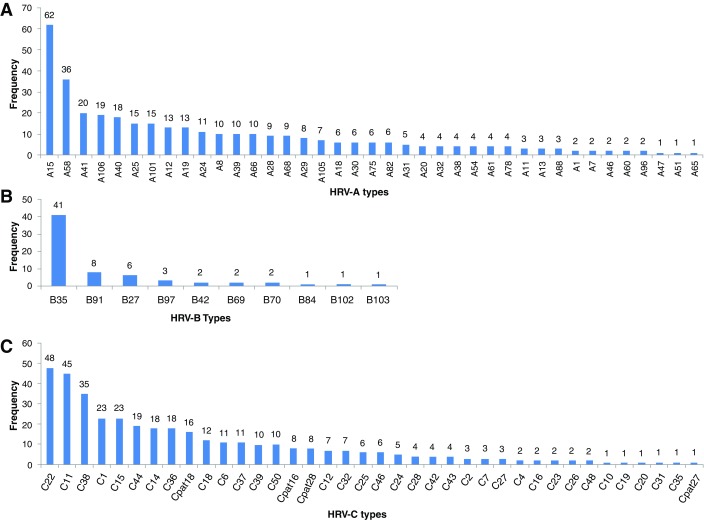
A Frequency distribution graph showing the human rhinovirus (HRV)-types detected in patient presenting to the nine health facilities within the KHDSS. ( **a**) HRV-A, ( **b**) HRV-B, ( **c**) HRV-C.

Phylogenetic analysis revealed 3 major clades representing the HRV-A, B and C species ( Supplementary Figure 1). Three samples were not assigned to any HRV type and these were identified as Enterovirus D68 (n=2) and Coxsackievirus B5 (n=1, see offshoot in Supplementary Figure 1). A total of 87 different HRV types were identified: 39 within species HRV-A, 10 within HRV-B and 38 within HRV- C. Within HRV-A, A15 was the predominant type (n=62), followed by A58 (n=36) and A41 (n=20). Other HRV-A types occurred at lower frequencies, ranging from 1 to 19 cases ( Figure 1 panel A). Within HRV-B, B35 presented as the dominant type (n=41) with the other HRV-B types being detected in 1 to 8 cases ( Figure 1 panel B). Within HRV-C, C22 type was the predominant type (n=48), followed closely by C11 (n=45) and C38 (n=35). The other HRV-C types were detected as shown in Figure 1 panel C.

**Figure 2.**
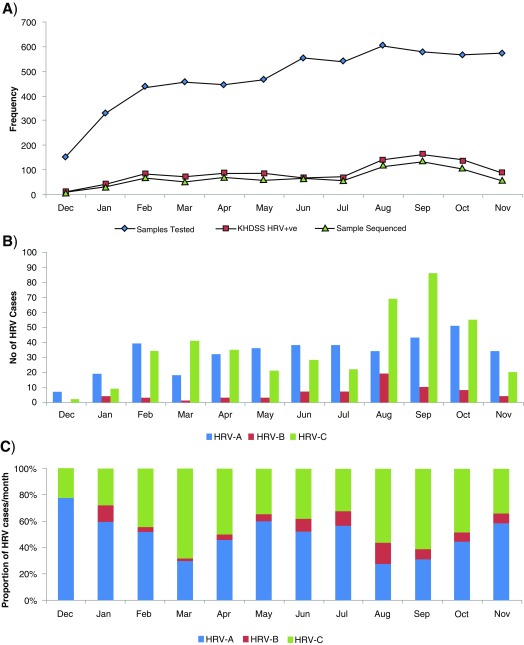
Temporal graph showing the distribution and proportions of human rhinovirus (HRV) species over a 12 month period detected in patient presenting to 9 health facilities within the Kilifi Health and Demographic Surveillance System (KHDSS) area, **a**) Frequency of total NPS samples collected, total HRV positives and total HRV positives sequenced, **b**) Monthly distribution of HRV species, **c**) Proportion of HRV-species observed over the 12 months period. HRV-A dominate in the months of December, January, February, May, June, July October and November while HRV-C dominated in the months of March, April, August and September.

HRV circulated throughout the study period as shown in Figure 2A. All HRV species were detected in all months except in December 2015 when only A and C were detected, although fewer samples were collected in this month. HRV incidence peaks were observed in August, September and October, while troughs were observed in December 2015 and January 2016, which was likely due to fewer samples collected in the first two months of the study. Despite HRV-C being the most prevalent, there was a varying dominance between HRV-A and HRV-C with time ( Figure 2B). HRV species A appeared dominant in December, January, February, May, June, July, October and November while HRV species C dominated in the months of March, April, August and September ( Figure 2C).

**Figure 3.**
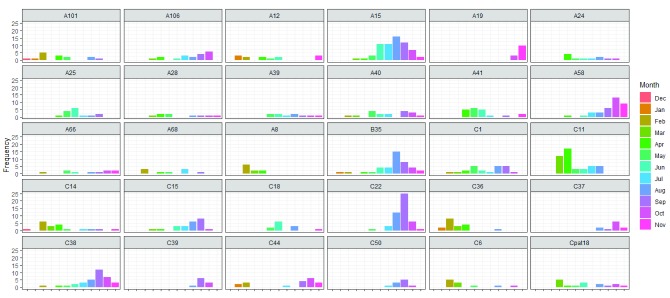
Temporal patterns by month of selected human rhinovirus (HRV) types obtained through ARI surveillance at nine health facilities in the Kilifi Health and Demographic Surveillance System, Kenya over the period December 2015 to November 2016.

Temporal patterns for each of the most frequently occurring types (with cases >16 during the year) are displayed in Figure 3. Most types displayed a unimodal distribution, with peak occurrence including August to October. However, there was considerable variation in the spread of the seasonal occurrence and modal month.

Regression analysis showed that the month of sample collection and age of participant were the most informative predictors for determining whether an individual tested positive for any HRV type. These predictors were identified whether undetermined samples were used in the regression analysis or not. Other factors considered were gender and health facility. First, five standard classification models (logistic regression, neural network using logistic activation functions and one hidden layer, k-nearest neighbor classification, tree and random forest regression) were compared using random partitions of the data into 2/3 training set, 1/3 testing set. Over 100-fold repetitions of training and testing each model, both logistic regression and the neural network classifiers had 77.2% classification accuracy (this increased to 81.0% and 80.9% respectively if undetermined samples were removed from the data set). Other classification models were less successful and because logistic regression is easier to interpret, and likelihood-based, we chose to continue our analysis using only logistic regression. Second, we removed uninformative predictor variables by comparing Akaike information criterion (AIC) for each additive combination of predictor variables and selecting the logistic regression model with lowest AIC. Whether undetermined samples were included in the regression analysis or not, in either case the lowest (i.e. best) AIC model included only month of sample collection and the age of the sample participant as predictors ( Supplementary Table 2, Supplementary File 1). The most informative models implied that the odds ratio of the participants being detected HRV positive declined by 0.1–0.2% per month of life, and that collection in August to October increased the odds ratio of finding HRV positives ( Supplementary Table 3, Supplementary File 1).

Notably, the health facility of sample collection was not included amongst the predictor variables for our classification analysis of HRV positive samples. However, this does not exclude the possibility of correlations in the types collected between different regions. Most sequences typed as belonging to the same HRV type were from different regions (represented by different health facilities) of the KHDSS and some were detected in several months. On the basis of phylogenetic analysis, closely related HRV-A15, C11, C22, C28 types were present in all 9 regions of KHDSS, and A66, B35, C14 and C44 types detected in 8 different regions. In other cases, identical HRV- types were shared in the different geographical regions ranging from 1 to 7 geographical regions. Nonetheless, the contingency table of type collected at each health facility ( Supplementary Table 4, Supplementary File 1) reveals highly significant evidence to reject independence of type occurrence at each health facility (G statistic = 849.7, dof = 704, P = 1.25×10 ^-4^) in favour of clustering of type occurrence by health facility.

**Figure 4.**
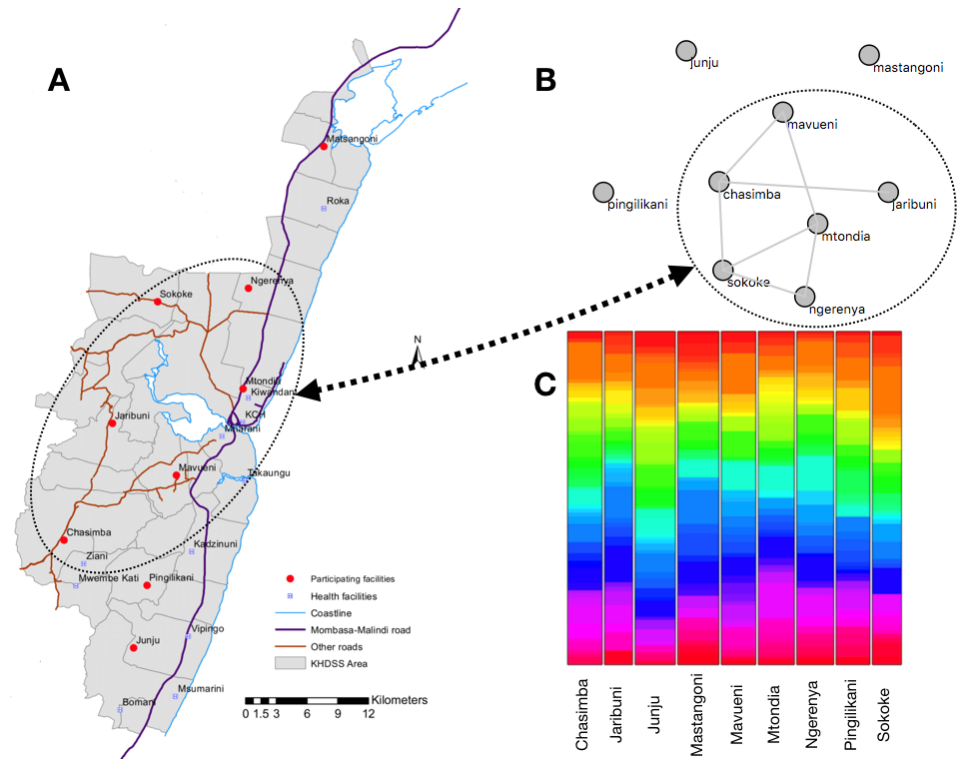
Type distribution similarity between health facilities in Kilifi Health and Demographic Surveillance System (KHDSS). **A**: Road map of KHDSS showing main highway (purple line), local roads (brown lines) and health facilities (red dots). **B**: Multidimensional scaling of the type distribution at each health facility along with pairwise similarity matrix. **C**: The type distribution at each health facility represented as a mosaic plot. The colors follow types in species human rhinovirus (HRV)-A (red, orange, yellow, green), HRV-B (light blue), and HRV-C (blue, dark blue, purple, red). Band widths are proportional to the number of each type sampled at the health facility.

In addition to clustering of individual type occurrence at the health facilities we also found evidence of greater similarity in HRV type distribution between the health facilities located near the road network in central Kilifi area (Chasimba, Jaribuni, Mavueni, Mtondia, Ngerenya, Sokoke) compared to the health facilities off the main road in the south of the KHDSS area (Junju, Pingilikani) and further north up the main highway (Matsangoni) ( Figure 4A). We measured the pairwise similarity of the type distribution at each health facility as the total absolute differences in their type occurrences shifted by the data median and rescaled by the median absolute distance from median (i.e. the rescaled Manhattan metric recommended for high dimensional data sets ^40^). We used the Orange implementation of MDS (see methods) to optimally represent the type distribution similarities on a plane along with a similarity network ( Figure 4B). The similarity network distinguishes between the type distributions of health facilities in the central part of Kilifi area on the road network, in particular the central similarity clique (Mtondia, Ngerenya, Sokoke), and the type distribution observed on the outskirts and harder to travel to parts of the Kilifi area. The MDS representation condenses and quantifies the information derived by direct visual comparison of the type distributions ( Figure 4C).

## Discussion

This study investigated the HRV infection, diversity and type distribution in individuals presenting with ARI symptoms for outpatient care at nine spatially structured health facilities in rural coastal Kenya over 12 months period. All three HRV species co-circulated, with HRV-A and HRV-C co-predominant. This observation agrees with a previous study conducted in rural coastal Kenya ^41^. Remarkably, a total of 87 HRV types were identified in circulation over the twelve months, representing 51.7% of all known HRV types. Occurrence of HRV throughout the year appears to be sustained by the existence of simultaneous and successive mini-epidemics each caused by a different HRV type introduced into the community independently. We assume each HRV type generates strong and lasting homotypic immunity, that leads to type-specific herd immunity and subsequent fadeout. This observation could be attributed to the natural ability of each type to independently cause an infection with limited cross-immunity with other types ^42^. Therefore, as long as there are new introductions and exposure to new HRV-types, the population experiences a series of new HRV infections throughout the year ^21– 23^.

As expected, there was a decrease in the detection of HRV cases with an increase in the age of the patients. The majority of the HRV positive patients were children below the age of 5 years (63.95%) and a statistically significant decrease in HRV positives amongst older ages was observed. The consistent finding of high proportions of HRV infections in children below the age of 5 years reiterates the need to focus control strategies to this age group since they are the most vulnerable, and presumably make the greatest contribution to community transmission. The low rates of detection of HRV types in adults may reflect the gradual development of type-specific immunity due to increased exposure throughout life.

We identified two Enterovirus D68s and one Coxsackievirus B5 in the nasopharyngeal samples initially classified as HRV positive using a real-time RT-PCR method. This is not unusual as the real time RT-PCR target region is genetically close between HRV and other members of the genus *Enterovirus* ^35^. A previous study conducted in Kilifi using the same molecular diagnostic assay reported a similar detection of non-HRV enteroviruses ^41^. Further, a recent study in Tanzania observed a relatively high prevalence of non-HRV enteroviruses in NPS sample including poliovirus type 1, enterovirus-D68, A71, echovirus-6, 7, 9 , 11 and a variety of coxsackievirus serotypes ^43^.

Phylogenetic analysis revealed close genetic association between sequences from different health facilities, with high intra-type genetic identities between the sequences from different health facilities (87 -100%). In some cases, the identical HRV-types were circulating simultaneously in different geographical regions separated by a distance as far as 30 km apart. Supported by the low genetic variation in the VP4/VP2 coding sequences and the concurrent distribution between these identical HRV-types it is probable that there is a rapid spread of HRV-types within the KHDSS once introduced and or many introductions of the same type into different areas of the KHDSS.

Despite phylogenetic evidence for rapid spread across the KHDSS, there is highly significant evidence for variation in the distribution of HRV-types between health facilities. An obvious mechanism that accounts for the variation in HRV-type distribution between health facilities is that transmission occurs more frequently between people attending the same health facility compared to those who attend other health facilities. In this case, sharing a health facility is a proxy for being more likely to live nearby and share the same social amenities and gatherings which are hotspot for transmission of respiratory viruses. Additionally, multidimensional scaling of the type distributions reveals greater type distribution similarity between the health facilities servicing parts of central Kilifi area that are more easily travelled between by road compared to the health facilities located significantly off the highway, or much further north along the highway. Spatial differentiation of type distribution, along with greater similarity in type distribution between the areas where we expect more human co-transit, is consistent with the expectations of spatial metapopulation models of infectious disease transmission ^44, 45^. However, it is comparatively rare to be able to demonstrate direct evidence of metapopulation effects in transmission, as we have done (see the discussion in Grenfell *et al*. ^46^).

We ascribed (above) the observation of multiple mini-epidemics in this community, each caused by a different HRV type, to the generation of homotypic herd immunity. If there was stronger heterotypic immunity, we would expect fewer HRV cases, and even periodic HRV fadeout. With strong homotypic and weak heterotypic immunity, we therefore expect newly introduced HRV types to spread unconstrained by pre-existing heterotypic immunity in the population. The differences in circulation periods observed among HRV-types could be as a result of stochastic differences in frequency of introduction and onward transmission of the HRV types, or variation in type-specific immunity. Similar observation has been reported in 47 where simultaneous and successive epidemics caused by different HRV types contributed to HRV high incidence and enabled HRV to remain in the local population for extended periods. In some cases, type-specific epidemics were served with different variants of the same HRV type probably as a result of separate introductions into the community at different times over the year. Moreover, comparing the HRV incidence with other respiratory viruses, as shown in the preceding study ^30^, HRV tend to peak in the second half of the year as other respiratory viruses report low incidence during this period. An explanation for this observation could be a case of virus interactions ^48, 49^ that whatever triggers (either environment or biological) high incidence in HRV in the 3 ^rd^ quarter of the year, causes low incidence in the other viruses. Further studies are needed to evaluate whether virus interaction affects incidence and transmission of individual viruses in the population.

Although the study has strength in its structured design and its implementation, it faced a number of limitations. First, there was low patient recruitment in the initial study months (December 2015 and January 2016) due to clinic closure on public holidays or people migrating to other areas of the country during festive season. This may have contributed to the lower observed HRV prevalence compared to the other months of study. Second, we failed to sequence the VP4/VP2 coding region for 22.2% of HRV positives samples, possibly due to low virus titers in the nasopharyngeal samples inferred from high Ct values (Ct value>33) in these samples. Third, since a maximum sample number per clinic was fixed, changes in prevalence of one virus, for example during a seasonal peak of coronavirus, would lead to fewer samples testing positive for other viruses. Hence there would be lack of independence of numbers of each virus type, or even between HRV types, over time, affecting temporal patterns of absolute numbers. However, the prevalence of each virus or virus type at any timepoint will reflect that circulating in the community.

In summary, we observed co-circulation of the three HRV species in all nine health facilities scattered in the KHDSS area of coastal Kenya, and combined we documented the circulation of a majority of all known HRV types to date (87/165) within a single year period in this small geographical area. Some of the HRV–types circulated in the KHDSS population close to all months of observation (10/12) suggesting marked local persistence of some types while others appeared and faded from circulation quite rapidly possibly due to low herd homologous immunity for the former and stronger herd homologous immunity in the latter. HRV transmission in the community is enhanced in people living close to one another and between areas linked by road network. Our study reports a substantial HRV burden among patients seeking outpatient care in this low-income setting of tropical Africa and a differential prevalence of the HRV species and types with significant differences in their local spatial-temporal distribution.

## Data availability

The replication data and analysis scripts for this manuscript are available from the Harvard Dataverse: https://doi.org/10.7910/DVN/DUQNDX ^50^.

As the dataset contains potentially identifying information on participants, it is stored under restricted access. Details on the eligibility for access and request form are available from http://kemri-wellcome.org/about-us/#ChildVerticalTab_15 for consideration by our Data Governance Committee ( dgc@kemri-wellcome.org)

## Supplementary material

Supplementary Figure 1: Phylogenetic analysis of 817 human rhinovirus (HRV) sequences from the Kilifi Health and Demographic Surveillance System (KHDSS) including 1 Coxsackievirus and 2 Enterovirus D68 sequences diagnosed as HRV by PCR. Different species are indicated by different tree branch color; black, HRV species A; red, HRV- species C; blue, HRV species B; pink, EV-D68; green, CV species B. HRV human rhinovirus, EV enterovirus, CV coxsackievirus.

Supplementary File 1: document containing the following Supplementary Tables:

Supplementary Table 1: Statistical comparison of clinical features and human rhinovirus (HRV) species.

Supplementary Table 2: Comparison of logistic regression models for identifying human rhinovirus (HRV) positive individuals.

Supplementary Table 3: Coefficients for best fitting logistic regression models.

Supplementary Table 4: Contingency table of sampled human rhinovirus (HRV)-types against collecting health facility.
