# Supplementary figures and images for: Human rhinovirus spatial-temporal epidemiology in rural coastal Kenya, 2015-2016, observed through outpatient surveillance
Source: Wellcome Open Res. 2019 Mar 27;3:128. Originally published 2018 Oct 1. [Version 2] doi: 10.12688/wellcomeopenres.14836.2 (PMC6234744; doi:10.12688/wellcomeopenres.14836.2)

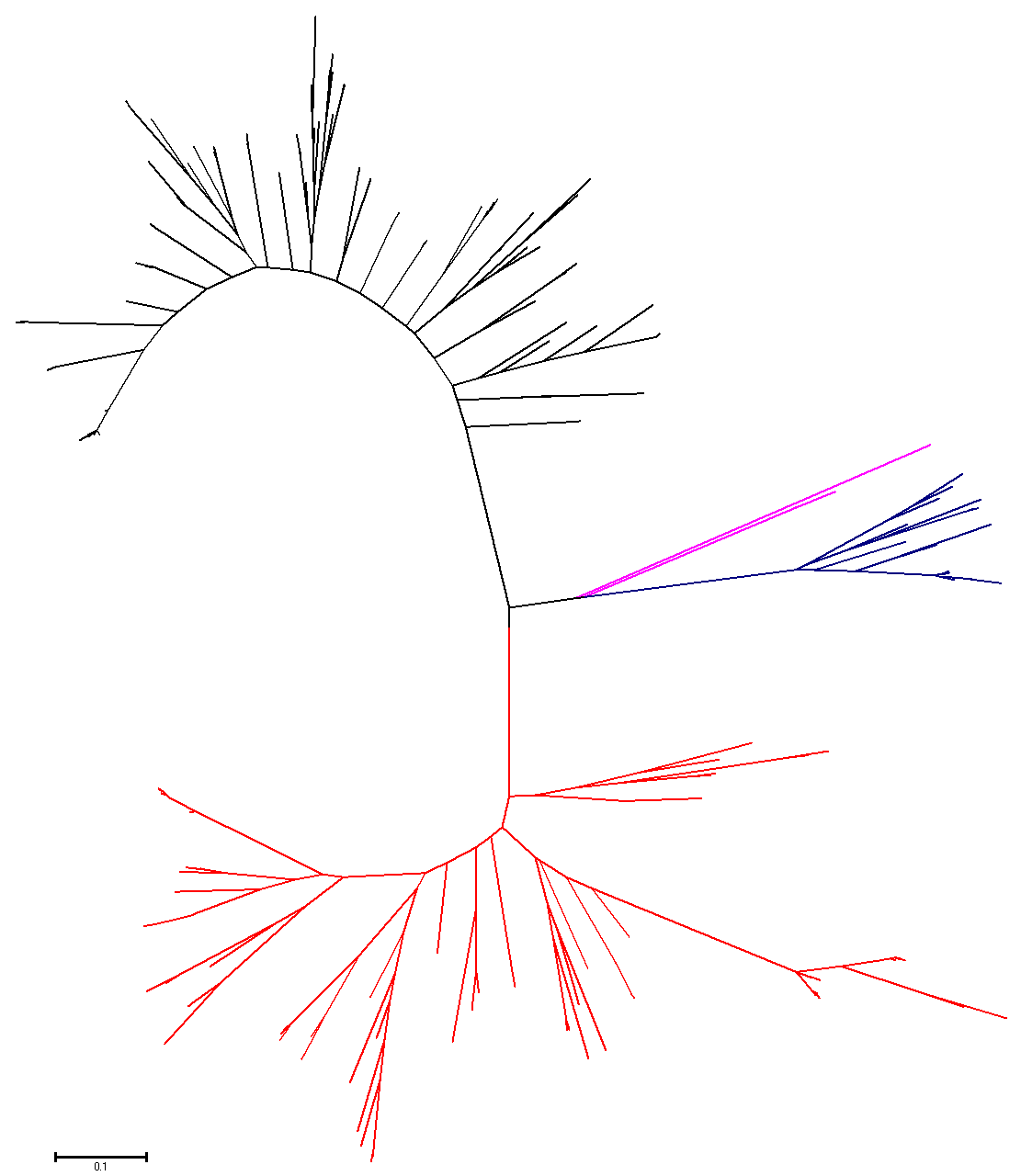

Supplement: Supplementary file 1 [file wellcomeopenres-3-16587-s0000.tgz › da5f2897-7f4b-4a88-ba23-0100812eec10_Supplementary_Figure_1.png]
