# Supplementary material for: Human rhinovirus spatial-temporal epidemiology in rural coastal Kenya, 2015-2016, observed through outpatient surveillance
Source: Wellcome Open Res. 2019 Mar 27;3:128. Originally published 2018 Oct 1. [Version 2] doi: 10.12688/wellcomeopenres.14836.2 (PMC6234744; doi:10.12688/wellcomeopenres.14836.2)
Supplement: Supplementary file 2 [file wellcomeopenres-3-16587-s0001.tgz › 89f0e93f-2103-4c0f-80ec-eb083557fb16_Supplementary_File_1_v2.docx]

Supplementary Table 1: Statistical comparison of clinical features and human rhinovirus (HRV) species.

|  | **Total HRV** | **HRV typed** | | | |  |  |
| --- | --- | --- | --- | --- | --- | --- | --- |
| **Clinical Features** | **n=1057** | Total typed  **817** | HRV-A  **360 (44.06)** | HRV-B  **67 (8.2)** | HRV-C  **390 (47.74)** | **P-value^1^** | **P-value^2^** |
| Cough | 999(94.51) | 773 (94.61) | 339(43.86) | 62(8.02) | 372(48.12) | 0.496 | 0.453 |
| Nasal Discharge | 845(79.94) | 654(80.04) | 305(46.64) | 51(7.80) | 298(45.57) | **0.012** | **0.004** |
| Difficulty in Breathing | 119(11.26) | 91(11.13) | 52(57.14) | 4(4.40) | 35(38.46) | **0.022** | **0.019** |
| Nasal Flaring | 22(2.08) | 17(2.08) | 5(29.41) | 1(5.88) | 11(64.71) | 0.386 | 0.175 |
| Sore throat | 64(6.05) | 52(6.36) | 26(50.00) | 3(5.77) | 23(44.23) | 0.723 | 0.463 |
| Chest-in drawing | 14(1.32) | 10(1.22) | 4(40.00) | 1(10.00) | 5(50.00) | 0.896 | 0.830 |
| Crackles | 14(1.32) | 11(1.34) | 4(36.36) | 1(9.09) | 6(54.55) | 0.814 | 0.610 |
| Wheeze | 28(2.65) | 23(2.82) | 7(30.43) | 3(13.04) | 13(56.52) | 0.303 | 0.238 |
| Lethargy | 8(0.75) | 5(0.61) | 3(60.00) | 1(20.00) | 1(20.00) | 0.201 | 0.279 |
|  |  |  |  |  |  |  |  |
| **Notes** | Data are no. (%) unless otherwise stated | | | | | |  |
|  | ^1^ chi square test of association/Fisher's exact test was used test the association between the clinical feature and HRV species. *P* values <0.05 were considered statistically significant (indicated in bold). | | | | | |  |
|  | ^2^ Test of proportions (between HRV species A and B) assuming equality | | | | | |  |

Supplementary Table 2: Comparison of logistic regression models for identifying human rhinovirus (HRV) positive individuals.

| **LOGISTIC REGRESSION: ‘undetermined’ samples included** | | |
| --- | --- | --- |
| **Model Formula** | **AIC** | **ΔAIC** |
| Pos ~ collection_month + age_months | 5327.809236 | 0 |
| Pos ~ sex + collection_month + age_months | 5328.543614 | 0.734377724 |
| Pos ~ facility + collection_month + age_months | 5334.638135 | 6.828898348 |
| Pos ~ sex +facility + collection_month + age_months | 5335.279524 | 7.47028724 |
| Pos ~ sex + collection_month | 5387.093179 | 59.28394221 |
| Pos ~ collection_month | 5393.170745 | 65.36150907 |
| Pos ~ sex + facility + collection_month | 5393.531299 | 65.72206257 |
| Pos ~ facility + collection_month | 5399.886071 | 72.07683481 |
| Pos ~ age_months | 5432.986192 | 105.1769557 |
| Pos ~ sex + age_months | 5433.56842 | 105.7591833 |
| Pos ~ facility + age_months | 5439.184117 | 111.3748803 |
| Pos ~ sex + facility + age_months | 5439.639624 | 111.8303881 |
| Pos ~ sex | 5483.265982 | 155.4567457 |
| Pos ~ sex + facility | 5488.958073 | 161.148837 |
| Pos ~ 1 | 5489.177222 | 161.367986 |
| Pos ~ facility | 5495.199054 | 167.3898181 |
| **LOGISTIC REGRESSION: ‘undetermined’ samples excluded** | | |
| **Model Formula** | **AIC** | **ΔAIC** |
| Pos ~ collection_month + age_months | 1270.653587 | 0 |
| Pos ~ facility + collection_month + age_months | 1271.405912 | 0.752324798 |
| Pos ~ sex + collection_month + age_months | 1271.987966 | 1.334379063 |
| Pos ~ sex +facility + collection_month + age_months | 1272.419003 | 1.765415333 |
| Pos ~ sex + collection_month | 1279.011793 | 8.358206225 |
| Pos ~ collection_month | 1279.258418 | 8.604831019 |
| Pos ~ sex + facility + collection_month | 1279.942236 | 9.28864828 |
| Pos ~ facility + collection_month | 1280.711597 | 10.05801023 |
| Pos ~ age_months | 1311.873757 | 41.22017 |
| Pos ~ sex + age_months | 1313.315579 | 42.66199143 |
| Pos ~ facility + age_months | 1314.100467 | 43.44687941 |
| Pos ~ sex + facility + age_months | 1315.348783 | 44.69519563 |
| Pos ~ 1 | 1317.055555 | 46.40196752 |
| Pos ~ sex | 1317.354061 | 46.70047364 |
| Pos ~ sex + facility | 1319.743244 | 49.08965661 |
| Pos ~ facility | 1319.752917 | 49.09932948 |

Supplementary Table 3: Coefficients for best fitting logistic regression models.

| **LOGISTIC REGRESSION: ‘undetermined ’ samples included** | | | |
| --- | --- | --- | --- |
| **Variable name** | **Coefficient** | **Standard Error** | **P value** |
| '(Intercept)' | -1.834280217 | 0.167775985 | 1.50E-27 |
| 'age_months' | -0.001584924 | 0.000210305 | 5.59E-14 |
| 'collection_month_2' | 0.537088379 | 0.204651411 | 0.00870303 |
| 'collection_month_3' | 0.329026035 | 0.210996118 | 0.118958365 |
| 'collection_month_4' | 0.588047157 | 0.205717552 | 0.0042716 |
| 'collection_month_5' | 0.541468046 | 0.205707991 | 0.008505691 |
| 'collection_month_6' | 0.015912661 | 0.212633533 | 0.940347741 |
| 'collection_month_7' | 0.150649737 | 0.211008398 | 0.475286288 |
| 'collection_month_8' | 0.893853259 | 0.193000262 | 3.71E-06 |
| 'collection_month_9' | 1.128514907 | 0.19144318 | 3.97E-09 |
| 'collection_month_10' | 0.940540413 | 0.194350844 | 1.34E-06 |
| 'collection_month_11' | 0.370770648 | 0.203579757 | 0.068620162 |
| 'collection_month_12' | -0.578468372 | 0.355091439 | 0.103353366 |
| **LOGISTIC REGRESSION: ‘undetermined ’ samples excluded** | | | |
| **Variable name** | **Coefficient** | **Standard Error** | **P value** |
| '(Intercept)' | 0.637613067 | 0.259408197 | 1.41E-02 |
| 'age_months' | -0.001229287 | 0.000366568 | 8.21E-04 |
| 'collection_month_2' | 0.547596614 | 0.333371057 | 0.100705632 |
| 'collection_month_3' | 0.412877383 | 0.341134714 | 0.226380505 |
| 'collection_month_4' | 0.845237317 | 0.351646652 | 0.01637073 |
| 'collection_month_5' | 0.554621694 | 0.334447016 | 0.097492111 |
| 'collection_month_6' | 1.134895847 | 0.399410837 | 0.004561023 |
| 'collection_month_7' | 1.686550005 | 0.454459217 | 0.000215024 |
| 'collection_month_8' | 1.372320986 | 0.34377029 | 6.92E-05 |
| 'collection_month_9' | 1.331414523 | 0.332268804 | 6.50E-05 |
| 'collection_month_10' | 2.006621795 | 0.398930229 | 5.59E-07 |
| 'collection_month_11' | 0.415769318 | 0.325227469 | 0.201337168 |
| 'collection_month_12' | -0.075309912 | 0.548614975 | 0.890836331 |

Supplementary Table 4: Contingency table of sampled human rhinovirus (HRV)-types against collecting health facility.

|  | **Health Facility** | | | | | | | | |
| --- | --- | --- | --- | --- | --- | --- | --- | --- | --- |
| **Type** | **Chasimba** | **Jaribuni** | **Junju** | **Matsangoni** | **Mavueni** | **Mtondia** | **Ngerenya** | **Pingilikani** | **Sokoke** |
| A1 | 0 | 0 | 0 | 1 | 0 | 0 | 0 | 0 | 1 |
| A101 | 2 | 0 | 5 | 3 | 3 | 1 | 0 | 1 | 0 |
| A105 | 0 | 1 | 0 | 4 | 0 | 1 | 1 | 0 | 0 |
| A106 | 1 | 2 | 2 | 2 | 3 | 2 | 1 | 2 | 4 |
| A11 | 0 | 2 | 0 | 0 | 0 | 0 | 0 | 0 | 1 |
| A12 | 0 | 0 | 1 | 2 | 0 | 3 | 1 | 4 | 2 |
| A13 | 0 | 0 | 2 | 1 | 0 | 0 | 0 | 0 | 0 |
| A15 | 10 | 4 | 8 | 3 | 11 | 4 | 7 | 6 | 11 |
| A18 | 1 | 2 | 0 | 0 | 0 | 0 | 2 | 0 | 1 |
| A19 | 0 | 0 | 5 | 3 | 0 | 1 | 0 | 2 | 2 |
| A20 | 0 | 0 | 1 | 1 | 0 | 1 | 1 | 0 | 0 |
| A24 | 1 | 1 | 0 | 3 | 2 | 0 | 3 | 0 | 1 |
| A25 | 1 | 1 | 3 | 0 | 2 | 0 | 1 | 6 | 1 |
| A28 | 2 | 0 | 1 | 0 | 1 | 2 | 2 | 0 | 1 |
| A29 | 0 | 3 | 1 | 0 | 0 | 2 | 1 | 0 | 1 |
| A30 | 1 | 0 | 0 | 1 | 1 | 1 | 1 | 0 | 1 |
| A31 | 0 | 0 | 1 | 0 | 0 | 2 | 0 | 2 | 0 |
| A32 | 0 | 2 | 0 | 1 | 0 | 1 | 0 | 0 | 0 |
| A38 | 1 | 0 | 0 | 1 | 0 | 2 | 0 | 0 | 0 |
| A39 | 3 | 1 | 2 | 1 | 0 | 1 | 1 | 1 | 0 |
| A40 | 1 | 0 | 3 | 2 | 4 | 2 | 2 | 4 | 0 |
| A41 | 3 | 2 | 6 | 0 | 0 | 4 | 0 | 2 | 3 |
| A46 | 0 | 0 | 1 | 0 | 0 | 1 | 0 | 0 | 0 |
| A47 | 1 | 0 | 0 | 0 | 0 | 0 | 0 | 0 | 0 |
| A51 | 0 | 1 | 0 | 0 | 0 | 0 | 0 | 0 | 0 |
| A54 | 2 | 0 | 1 | 1 | 0 | 0 | 0 | 0 | 0 |
| A58 | 4 | 2 | 6 | 5 | 7 | 2 | 5 | 3 | 2 |
| A60 | 0 | 0 | 0 | 0 | 0 | 0 | 1 | 0 | 1 |
| A61 | 1 | 0 | 1 | 2 | 0 | 0 | 0 | 0 | 0 |
| A65 | 0 | 0 | 0 | 0 | 0 | 0 | 0 | 0 | 1 |
| A66 | 2 | 1 | 1 | 1 | 0 | 1 | 1 | 2 | 1 |
| A68 | 0 | 0 | 4 | 0 | 0 | 1 | 1 | 1 | 2 |
| A7 | 0 | 0 | 0 | 0 | 0 | 1 | 1 | 0 | 0 |
| A75 | 2 | 1 | 0 | 0 | 1 | 1 | 0 | 1 | 0 |
| A78 | 1 | 0 | 1 | 0 | 0 | 0 | 2 | 0 | 0 |
| A8 | 2 | 1 | 0 | 1 | 0 | 0 | 0 | 6 | 0 |
| A82 | 0 | 0 | 0 | 2 | 0 | 1 | 1 | 0 | 2 |
| A88 | 0 | 0 | 0 | 0 | 0 | 0 | 0 | 2 | 1 |
| A96 | 0 | 1 | 0 | 0 | 0 | 0 | 0 | 0 | 1 |
| B102 | 0 | 0 | 0 | 0 | 0 | 0 | 0 | 1 | 0 |
| B103 | 1 | 0 | 0 | 0 | 0 | 0 | 0 | 0 | 0 |
| B27 | 0 | 0 | 3 | 1 | 1 | 0 | 0 | 0 | 1 |
| B35 | 4 | 1 | 4 | 5 | 7 | 7 | 10 | 3 | 0 |
| B42 | 0 | 0 | 2 | 0 | 0 | 0 | 0 | 0 | 0 |
| B69 | 0 | 0 | 0 | 0 | 0 | 2 | 0 | 0 | 0 |
| B70 | 1 | 0 | 0 | 0 | 0 | 0 | 0 | 0 | 1 |
| B84 | 0 | 0 | 1 | 0 | 0 | 0 | 0 | 0 | 0 |
| B91 | 1 | 1 | 1 | 0 | 0 | 0 | 4 | 0 | 1 |
| B97 | 0 | 1 | 0 | 0 | 2 | 0 | 0 | 0 | 0 |
| C | 1 | 2 | 2 | 1 | 1 | 0 | 0 | 0 | 0 |
| C1 | 2 | 1 | 1 | 3 | 4 | 3 | 5 | 0 | 4 |
| C10 | 0 | 0 | 0 | 0 | 0 | 0 | 1 | 0 | 0 |
| C11 | 5 | 7 | 6 | 11 | 3 | 2 | 3 | 4 | 4 |
| C12 | 0 | 1 | 2 | 0 | 2 | 0 | 0 | 2 | 0 |
| C14 | 0 | 1 | 3 | 3 | 2 | 2 | 3 | 3 | 1 |
| C15 | 3 | 2 | 0 | 7 | 3 | 2 | 2 | 3 | 1 |
| C16 | 0 | 0 | 0 | 0 | 0 | 1 | 0 | 1 | 0 |
| C18 | 2 | 2 | 3 | 2 | 1 | 1 | 0 | 1 | 0 |
| C19 | 0 | 0 | 1 | 0 | 0 | 0 | 0 | 0 | 0 |
| C2 | 1 | 0 | 0 | 0 | 0 | 0 | 1 | 1 | 0 |
| C20 | 1 | 0 | 0 | 0 | 0 | 0 | 0 | 0 | 0 |
| C22 | 4 | 8 | 5 | 7 | 5 | 6 | 7 | 0 | 6 |
| C23 | 2 | 0 | 0 | 0 | 0 | 0 | 0 | 0 | 0 |
| C24 | 0 | 0 | 0 | 2 | 2 | 0 | 0 | 1 | 0 |
| C25 | 0 | 0 | 0 | 2 | 0 | 2 | 1 | 1 | 0 |
| C26 | 0 | 0 | 1 | 0 | 0 | 0 | 1 | 0 | 0 |
| C27 | 0 | 0 | 0 | 0 | 3 | 0 | 0 | 0 | 0 |
| C28 | 0 | 1 | 0 | 0 | 2 | 1 | 0 | 0 | 0 |
| C31 | 0 | 0 | 0 | 0 | 0 | 1 | 0 | 0 | 0 |
| C32 | 0 | 1 | 3 | 0 | 1 | 0 | 0 | 2 | 0 |
| C35 | 1 | 0 | 0 | 0 | 0 | 0 | 0 | 0 | 0 |
| C36 | 1 | 3 | 0 | 4 | 4 | 3 | 2 | 1 | 0 |
| C37 | 2 | 0 | 4 | 0 | 1 | 0 | 0 | 4 | 0 |
| C38 | 5 | 4 | 1 | 3 | 1 | 10 | 6 | 1 | 4 |
| C39 | 4 | 2 | 0 | 0 | 0 | 0 | 1 | 2 | 1 |
| C4 | 0 | 0 | 0 | 0 | 0 | 0 | 2 | 0 | 0 |
| C42 | 0 | 0 | 0 | 0 | 0 | 0 | 0 | 2 | 2 |
| C43 | 1 | 0 | 0 | 0 | 1 | 1 | 0 | 0 | 1 |
| C44 | 2 | 4 | 2 | 1 | 4 | 3 | 2 | 0 | 1 |
| C46 | 0 | 0 | 0 | 3 | 1 | 2 | 0 | 0 | 0 |
| C48 | 1 | 0 | 0 | 0 | 0 | 1 | 0 | 0 | 0 |
| C50 | 0 | 0 | 1 | 3 | 1 | 1 | 2 | 2 | 0 |
| C6 | 1 | 0 | 1 | 0 | 1 | 2 | 2 | 3 | 1 |
| C7 | 0 | 0 | 1 | 1 | 0 | 1 | 0 | 0 | 0 |
| C_pat18 | 2 | 0 | 0 | 1 | 0 | 0 | 2 | 3 | 1 |
| C_pat27 | 0 | 0 | 0 | 0 | 0 | 0 | 1 | 0 | 0 |
| Cpat16 | 0 | 0 | 1 | 2 | 1 | 1 | 0 | 0 | 3 |
| Cpat18 | 0 | 3 | 0 | 1 | 0 | 1 | 0 | 0 | 2 |
| Cpat28 | 0 | 0 | 0 | 3 | 1 | 0 | 2 | 2 | 0 |
