# Supplementary material for: Human rhinovirus spatial-temporal epidemiology in rural coastal Kenya, 2015-2016, observed through outpatient surveillance
Source: Wellcome Open Res. 2019 Mar 27;3:128. Originally published 2018 Oct 1. [Version 2] doi: 10.12688/wellcomeopenres.14836.2 (PMC6234744; doi:10.12688/wellcomeopenres.14836.2)
Supplement: Peer review history

# Peer review: Reviewer response for version 2

**DOI:** 10.21956/wellcomeopenres.16587.r35149

**Published:** 2019-03-29

**Authors:** Ian M. Mackay

**Affiliations:** 1 Faculty of Medicine, Child Health Research Centre, The University of Queensland, Brisbane, Qld, Australia

Great work.

I have read this submission. I believe that I have an appropriate level of expertise to confirm that it is of an acceptable scientific standard.

# Peer review: Reviewer response for version 1

**DOI:** 10.21956/wellcomeopenres.16165.r34078

**Published:** 2018-11-12

**Authors:** Mbayame Ndiaye Niang

**Affiliations:** 1 Medical Virology Unit, Institut Pasteur Dakar, Dakar, Senegal

The authors have determined human rhinovirus spatial-temporal epidemiology in rural coastal Kenya, observed through outpatient surveillance. This study was conducted over 11 months and showed a high prevalence and diversity of HRV in out-patient with symptoms of acute respiratory infection.

 Globally, the paper is very interesting because it allows not only to know the different serotypes of rhinovirus circulating in Kenya but also their spatial-temporal circulation. The study is also a contribution to the scarcity of data observed in low-income settings regarding HRV circulation and diversity.

 Some remarks and comments about the study are noted:

**Study design**: The case definition used should be more specific and explain why children under 7 are excluded from the study. A more appropriate reference for acute respiratory infections should be used (WHO Guideline describing standards for influenza surveillance including case definitions for ILI and SARI ^1^).

 Authors should specify if the sample is a nasopharyngeal or nasal specimen swab. The cited technique appears to be a nasal swab and not a nasopharyngeal swab.

**Laboratory procedures**

 Why are samples with Ct> 35 considered negative? This was not explained in reference 27, which the author refers to us.

 The multiplex real-time PCR assay system used for the detection of  rhinoviruses does not differentiate between rhinoviruses and enteroviruses. Other enteroviruses such as Cox B5 and EV-D68 have been found, and this may lead to overestimation of the prevalence of rhinoviruses

**Discussion**

 Remarkably, a total of 87 HRV types were identified in circulation over the twelve months, representing 52.7% of all known HRV types: Could the authors provide the total number of HRV types to better understand how the 52.7% were obtained

 Other members of the genus *Enterovirus *was identified: the authors could discuss the use of more specific detection methods for rhinovirus than to mention similar studies. This part must be more discussed.

 The authors mention a predominance of rhinovirus C followed by rhinovirus A: authors can discuss this predominance by analyzing the clinical signs of the study population. It is known that rhino C is more prevalent in asthmatics.

 When discussing occurrence of multiple typical mini-epidemics caused by specific types in the community, the authors should refer to other studies made elsewhere and add these references to the discussion.

I have read this submission. I believe that I have an appropriate level of expertise to confirm that it is of an acceptable scientific standard.

## References

[1] World Health Organisation: Global epidemiological surveillance standards for influenza. *World Health Organisation*.2014;

# Response

**Published:** 2019-03-20

**Authors:** john mwita

**Affiliations:** JKUAT, Kenya

**The authors have determined human rhinovirus spatial-temporal epidemiology in rural coastal Kenya, observed through outpatient surveillance. This study was conducted over 11 months and showed a high prevalence and diversity of HRV in out-patient with symptoms of acute respiratory infection.**

**Response**

We would like to clarify that the study was carried out over a period of 12 months (December 2015 to November 2016, inclusive). In the section on Study design we have added the word “inclusive” to prevent uncertainty.

**Globally, the paper is very interesting because it allows not only to know the different serotypes of rhinovirus circulating in Kenya but also their spatial-temporal circulation. The study is also a contribution to the scarcity of data observed in low-income settings regarding HRV circulation and diversity.**

**Response**

We thank the reviewer for these comments.

**The case definition used should be more specific and explain why children under 7 are excluded from the study. A more appropriate reference for acute respiratory infections should be used (WHO Guideline describing standards for influenza surveillance including case definitions for ILI and SARI1).**

**Response**

All details of eligibility and sample collection are described in the baseline report on the study (Nyiro et al. 2018). Individuals of any age were eligible excepting those aged less than 7 days. This exclusion was made since it was believed few children less than 7 days of age would present with respiratory virus infections and considered that subjecting the child to a deep NPS was not justified. We did not use ILI or SARI definitions, instead we used cough, sneezing, nasal congestion, difficulty in breathing, or fast breathing for age (the latter as defined by WHO guidelines on patient management of common childhood illnesses (2013).

**Authors should specify if the sample is a nasopharyngeal or nasal specimen swab. The cited technique appears to be a nasal swab and not a nasopharyngeal swab.**

**Response**

A deep nasopharyngeal swab was collected from participants, as described in Nyiro et al 2018. For clarity we have added the text “to a distance where the tip located the deep nasopharynx,” in the last paragraph of Study design.

**Why are samples with Ct> 35 considered negative? This was not explained in reference 27, which the author refers to us.**

**Response**

The reviewer is correct that the reason for considering as negative samples with Ct>=35.0 was not explained in ref 27. We apologize for this oversight. The cut-off value of Ct 35.0 was given in the paper describing the protocol that this study followed (Hammitt et al. 2011),  but the rationale for setting the cut-off at 35.0 was not given. The reference for this paper has now been added to the manuscript on Laboratory procedures section. In addition, experience has shown us that VP4/VP2 RT-PCR often (95%) fails for samples with Ct > 35.0.

**The multiplex real-time PCR assay system used for the detection of rhinoviruses does not differentiate between rhinoviruses and enteroviruses. Other enteroviruses such as Cox B5 and EV-D68 have been found, and this may lead to overestimation of the prevalence of rhinoviruses**

**Response**

There is potential for over-estimation of rhinovirus prevalence for the reason stated.HRV and other enteroviruses are closely related at the genetic level. However, we sequenced in the VP2/VP4 region 78% of the rhinovirus positives (817/1057), identifying only 3 non-rhinovirus enteroviruses. This suggests that the over-estimation of prevalence is small.

**Remarkably, a total of 87 HRV types were identified in circulation over the twelve months, representing 52.7% of all known HRV types: Could the authors provide the total number of HRV types to better understand how the 52.7% were obtained**

**Response**

Current HRV classification method puts the total number of HRV types at 168, www.picornaviridae.com, (accessed on 13 February 2019). Our study identified and typed 87 HRV types: these represent 51.7% of the known HRVs. This has been discussed in the first paragraph of Study design.

**Other members of the genus Enterovirus was identified: the authors could discuss the use of more specific detection methods for rhinovirus than to mention similar studies. This part must be more discussed.**

**Response**

We are not entirely sure what the reviewer is requesting. What we can say is that the study used a sensitive real-time PCR for the detection of HRV. Further that the assay targeted the 5’ UTR which is genetically similar for both rhinoviruses and other enteroviruses. As a result, non-rhinovirus enteroviruses were detected. However, these were uncommon. Using primers that anneal to only the highly conserved region on the 5 ‘UTR of all the HRV species can provide the most sensitive assay for the detection of HRV strains

(Bochkov et al. 2014)

**The authors mention a predominance of rhinovirus C followed by rhinovirus A: authors can discuss this predominance by analyzing the clinical signs of the study population. It is known that rhino C is more prevalent in asthmatics.**

**Response**

Based on test of proportions assuming equality, there was no significant (p < 0.05) difference between species A and C (predominant species) for the clinical signs reported except for nasal discharge (p< 0.0042) and difficult breathing (p<0.0194), which differed significantly. We have added this information on the second paragraph of Results section and also inserted a column on the same in supplementary Table 1

**When discussing occurrence of multiple typical mini-epidemics caused but specific types in the community, the authors should refer to other studies elsewhere and add these references to the discussion.**

**Response**

We are aware of one other study showing multiple typical mini-epidemics (Sansone et al. 2013).The study demonstrates that simultaneous and successive epidemics caused by different HRV types contributed to high HRV incidence and enabled HRV to remain in the local population for extended periods. We have added text in the Discussion section (page14) to this effect.

**References**

Bochkov, Yury A, Kristine Grindle, Fue Vang, Michael D Evans, and James E Gern. 2014. “Improved Molecular Typing Assay for Rhinovirus Species A, B, and C.” *Journal of Clinical Microbiology* 52 (7): 2461–71. https://doi.org/10.1128/JCM.00075-14.

Hammitt, Laura L, Sidi Kazungu, Steve Welch, Anne Bett, Clayton O Onyango, Rory N Gunson, J Anthony G Scott, and D James Nokes. 2011. “Added Value of an Oropharyngeal Swab in Detection of Viruses in Children  Hospitalized with Lower Respiratory Tract Infection.” *Journal of Clinical Microbiology* 49 (6): 2318–20. https://doi.org/10.1128/JCM.02605-10.

Nyiro, Joyce Uchi, Patrick Munywoki, Everlyn Kamau, Charles Agoti, Alex Gichuki, Timothy Etyang, Grieven Otieno, and D James Nokes. 2018. “Surveillance of Respiratory Viruses in the Outpatient Setting in Rural Coastal Kenya: Baseline Epidemiological Observations.” *Wellcome Open Research* 3: 89. https://doi.org/10.12688/wellcomeopenres.14662.1.

Sansone, M, M Andersson, R Brittain-Long, L-M Andersson, S Olofsson, J Westin, and M Lindh. 2013. “Rhinovirus Infections in Western Sweden: A Four-Year Molecular Epidemiology Study Comparing Local and Globally Appearing Types.” *European Journal of Clinical Microbiology & Infectious Diseases* *: Official Publication of the European Society of Clinical Microbiology* 32 (7): 947–54. https://doi.org/10.1007/s10096-013-1832-x.

# Peer review: Reviewer response for version 1

**DOI:** 10.21956/wellcomeopenres.16165.r34035

**Published:** 2018-10-16

**Authors:** Ian M. Mackay

**Affiliations:** 1 Faculty of Medicine, Child Health Research Centre, The University of Queensland, Brisbane, Qld, Australia

The study is a nice examination of the complexity and scope of RV circulation. It adds to an established but growing literature acknowledging that “rhinovirus” is not one virus, but a large group of distinct viruses capable of apparently independent co-circulation. This circulation includes the majority of genotypes (types) at a single location over time using molecular methods augmented with sequencing. The paper notes some important aspects for further study.

 Some questions and comments about the study include:

1. Introduction: RV infections can also be mild and asymptomatic.
2. A good reference to the naming of 160 genotypes for your readers, is www.picornavirade.com
3. The authors make mention of RV studies in nations being rare and that this study’s area has a tropical climate. ollow-up on these and putting the study findings into context in the discussion would be helpful
4. Could the authors add some information about why they excluded infants younger than days and people with ARI symptoms for longer than 30 days?
5. The authors note that their genotyping process is “as previously described”. But it is quite different including not being nested RT-PCR (which can enhance sensitivity). Could they provide more detail – perhaps consider adding their method in complete detail to https://www.protocols.io/ and referencing to that in this paper.
6. Could the authors better justify their choice to use a 35 Ct threshold for PCR Also–please check that the reference used is correct.
7. Did the evidence 300nt was detrimental to calling genotypes? For example, they use truncated longer amplicons and the outcome by BLAST to determine a real difference?
8. The authors might consider the term “species ” to describe the “varying dominance” between HRV species discussed around Figure 2.
9. Could the authors add a comment about circulation of RVs throughout the year and the role this might play, if it occurs, in ‘’ of the same or different RV
10. Could the add some referenced to pre-existing knowledge where discussion comments have about (a) of heterotypic immunity to RVs and (b) virus interactions?
11. In their summary, the authors imply that the patterns in genotypes and species exchange detected in the nine health facilities are the same as those in the community. How sure can they be that there is not a mild and asymptomatic non-presenting infections?
12. Could the authors expand on their 22.2% VP4/VP2 sequencing failure in the context of the comment about changed methods above?

I have read this submission. I believe that I have an appropriate level of expertise to confirm that it is of an acceptable scientific standard.

# Response

**Published:** 2019-03-20

**Authors:** john mwita

**Affiliations:** JKUAT, Kenya

**The study is a nice examination of the complexity and scope of RV circulation. It adds to an established but growing literature acknowledging that “rhinovirus” is not one virus, but a large group of distinct viruses capable of apparently independent co-circulation. This circulation includes the majority of genotypes (types) at a single location over time using molecular methods augmented with sequencing. The paper notes some important aspects for further study.**

**Response**

We thank the reviewer for these comments.

**Introduction: RV infections can also be mild and asymptomatic**

**Response**

We have added text at the first paragraph on Introduction section to reflect human rhinoviruses infections not only present as severe respiratory tract infections but can also present as mild and asymptomatic.

**A good reference to the naming of 160 genotypes for your readers, is** *www.picornavirade.com*

**Response**

Thank you for this suggestion. We have added the number so far identified (168) and the reference to the URL suggested and in the Methods section on Sequence alignment.

**The authors make mention of RV studies in nations being rare and that this study’s area has a tropical climate. Follow-up on these and putting the study findings into context in the discussion would be helpful**.

**Response**

HRV in sub Saharan Africa is not well described although some studies on molecular epidemiology of HRV species among the populations have revealed substantial diversity of HRV types in this region (L’Huillier et al. 2015)(Milanoi et al. 2016). The present study not only identifies the HRV types in circulation but describes their spatial-temporal patterns in the community. These data provide added perspectives on the spread patterns of HRV types in a community.

**Could the authors add some information about why they excluded infants younger than 7 days and people with ARI symptoms for longer than 30 days?**

**Response**

We provided an explanation for the 7day exclusion in response to question 3 by the first reviewer. We felt that the inclusion of this age group would not significantly increase cases (of all respiratory viruses) and hence considered the collection of a deep NPS could not be justified. Viral ARI are self-limiting, often lasting between 7-14 days (Pappas et al. 2008) for immune-competent individuals. For persistent ARI (>14 days), the individual could be having other underlying chronic infections e.g. TB or immune compromised due HIV-AIDS or malignancy (Chu et al. 2016)(Piralla et al. 2015).

**The authors note that their genotyping process is “as previously described”. But it is quite different including not being nested RT-PCR (which can enhance sensitivity). Could they provide more detail – perhaps consider adding their method in complete detail to https://www.protocols.io/ and referencing to that in this paper.**

**Response**

Thank you for pointing this out as the method was not a nested RT-PCR. We apologize for this oversight. The RT-PCR method used to amplify the VP4/VP2 genomic region was a modification of a previous protocol (Wisdom et al. 2009). Forward primer OS (CCGGCCCCTGAATGYGGCTAA) and reverse primer IAS (TCWGGHARYTTCCAMCACCANCC) were used in a RT-PCR reaction using Qiagen OneStep RT-PCR kit. Thermocycling conditions were set as follows:  Reverse transcription of the RNA to cDNA at 50 ^o^C for 30 minutes, in-activation of reverse transcriptase and activation of Taq polymerase at 95 ^o^C for 15 minutes. This was followed by 40 cycles of denaturation at 94 ^o^C for 1 minute, annealing at 60 ^o^C for 1 minute and strand extension at 72 ^o^C for 1 min and final incubation/extension at 72 ^o^C for 10 minutes. We have added text in the Laboratory procedures section to this effect.

**Could the authors better justify their choice to use a 35 Ct threshold for PCR Also–please check that the reference used is correct.**

**Response**

We provided an explanation for the use of 35 Ct threshold for PCR in response to question 5 by the first reviewer. Ostensibly, the sequencing success reduced considerably for samples with Ct values >35.0 (i.e. for samples with low viral load). 

**Did the evidence 300nt was detrimental to calling genotypes? For example, they use truncated longer amplicons and the outcome by BLAST to determine a real difference?**

**Response**

Indeed, shorter sequences were detrimental to calling genotypes, we had to set 300nt of the VP4/VP2 region as the minimum nucleotide size to call HRV types because, with reduction of nucleotide size a typed sequence tends to shift to other branches of genotype/type or fails to cluster with a reference sequence thus falling as untypable.

**The authors might consider the term “species” to describe the varying dominance between HRV species discussed around Figure 2.**

**Response**

We are not entirely clear what the reviewer is suggesting. However, we have now added “species” in the text describing the varying dominance between HRV species-A and C in the first paragraph on page 10 of the manuscript.

**Could the authors add a comment about circulation of RVs throughout the year and the role this might play, if it occurs, in ‘’ of the same or different RV**

**Response**

The circulation of HRV throughout the year appears to be maintained by the existence of simultaneous and successive mini-epidemics each caused by a different HRV type introduced into the community independently. We assume that each HRV type generates strong and lasting homotypic immunity, that leads to type-specific herd immunity and fade out. We have added text in the first paragraph of Discussion section.

**Could the author add some referenced to pre-existing knowledge where discussion comments have about (a) of heterotypic immunity to RVs and (b) virus interactions?**

**Responses**

Heterotypic immunity against HRV infections is generally weak and short-term (lasting between 5 weeks and 16 weeks)(Fleet et al. 1965, 1968; Stott et al. 1969) compared to strong homotypic immunity (~1 year)(Barclay et al. 1989). Although heterotypic immunity can be established it is not significant to protect an individual from other multiple HRV types (Stott et al. 1969). The duration of homotypic immunity may be enhanced by reinfection with other types due to non-specific boosting of titers of types to which individuals were previously exposed(Fleet et al. 1965). Our own recent study of household HRV infection revealed rapid heterotypic reinfection in younger individuals (e.g. the median time to reinfection in 1-10 year olds was 1month), with a decreasing rate in older individuals (Kamau et al. 2018) who presumably had past experience of more types and hence reduced susceptibility.

We have also added references (Mackay 2008; Brunstein et al. 2008) in the Discussion section (page 14), which comments on virus interactions. Mackay's review of human rhinoviruses (Mackay 2008) indicates that virus interactions could be the cause of seasonal variation in the prevalence of any virus, whereby the high prevalence of one respiratory virus moderates or prevents processes that allow one or more other viruses to establish themselves in the host at the same time. The review also suggests that HRV partnership with host immunity can be mutual, incidentally giving the host an advantage by protecting against more cytopathic viral pathogens in the respiratory system while the host provides a vessel for HRV replication and transmission.

**In their summary, the authors imply that the patterns in genotypes and species exchange detected in the nine health facilities are the same as those in the community. How sure can they be that there is not a mild and asymptomatic non-presenting infection?**

**Response**

The reviewer is right, we might have missed the mild and asymptomatic non-presenting infections since we only sampled from patients who presented to health centers with symptoms of ARI. However, we believe our surveillance is representative of circulation of all HRV infections because (a) we have a representative spatial distribution of health facilities and (b) our study of the household where we collected samples irrespective of symptoms showed  similar type distributions for symptomatic and asymptomatic individuals (Kamau et al. 2018) .

**Could the authors expand on their 22.2% VP4/VP2 sequencing failure in the context of the comment about changed methods above**

**Response**

We are not certain of the reasons for the 22.7% failure (240 samples) in sequencing. Viral load was slightly lower for failed samples: the mean Ct value for sequenced versus failed samples was 29.7 (standard deviation 3.1, 817 samples) and 30.7 (standard deviation 3.2, 240 samples) respectively. Furthermore, we had 154/240 samples failed at the PCR stage, which could have resulted from mismatches in the primer binding sites or assay sensitivity given that we were using a single step rather than nested PCR.

**References**

Barclay, W S, W Al-Nakib, P G Higgins, and D A J Tyrrell. 1989. “The Time Course of the Humoral Immune Response to Rhinovirus Infection.” *Epidemiology & Infection* 103 (3): 659–69.

Brunstein, John D, Christy L Cline, Steven McKinney, and Eva Thomas. 2008. “Evidence from Multiplex Molecular Assays for Complex Multipathogen Interactions in Acute Respiratory Infections.” *Journal of Clinical Microbiology* 46 (1): 97 LP-102. https://doi.org/10.1128/JCM.01117-07.

Chu, Helen Y, Jennifer Chin, Jessica Pollard, Danielle M Zerr, and Janet A Englund. 2016. “Clinical Outcomes in Outpatient Respiratory Syncytial Virus Infection in Immunocompromised Children.” *Influenza and Other Respiratory Viruses* 10 (3): 205–10. https://doi.org/10.1111/irv.12375.

Fleet, W F, R B Couch, T R Cate, and V Knight. 1965. “Homologous and Heterologous Resistance to Rhinovirus Common Cold.” *American Journal of Epidemiology* 82 (2): 185–96.

Fleet, W F, R G Jr Douglas, T R Cate, and R B Couch. 1968. “Antibody to Rhinovirus in Human Sera. II. Heterotypic Responses.” *Proceedings of the Society for Experimental Biology and Medicine. Society for Experimental Biology and Medicine (New York, N.Y.)* 127 (2): 503–9.

Kamau, Everlyn, Clayton O Onyango, Grieven P Otieno, Patience K Kiyuka, Charles N Agoti, Graham F Medley, Patricia A Cane, D James Nokes, and Patrick K Munywoki. 2018. “An Intensive, Active Surveillance Reveals Continuous Invasion and High Diversity of Rhinovirus in Households,” December. https://doi.org/10.1093/infdis/jiy621.

L’Huillier, Arnaud G, Laurent Kaiser, Tom J Petty, Mary Kilowoko, Esther Kyungu, Philipina Hongoa, Gaël Vieille, et al. 2015. “Molecular Epidemiology of Human Rhinoviruses and Enteroviruses Highlights Their  Diversity in Sub-Saharan Africa.” *Viruses* 7 (12): 6412–23. https://doi.org/10.3390/v7122948.

Mackay, Ian M. 2008. “Human Rhinoviruses: The Cold Wars Resume.” *Journal of Clinical Virology* *: The Official Publication of the Pan American Society for Clinical Virology* 42 (4): 297–320. https://doi.org/10.1016/j.jcv.2008.04.002.

Milanoi, Sylvia, Juliette R Ongus, George Gachara, Rodney Coldren, and Wallace Bulimo. 2016. “Serotype and Genetic Diversity of Human Rhinovirus Strains That Circulated in Kenya  in 2008.” *Influenza and Other Respiratory Viruses* 10 (3): 185–91. https://doi.org/10.1111/irv.12373.

Pappas, Diane E, J Owen Hendley, Frederick G Hayden, and Birgit Winther. 2008. “Symptom Profile of Common Colds in School-Aged Children.” *The Pediatric Infectious Disease Journal* 27 (1): 8–11. https://doi.org/10.1097/INF.0b013e31814847d9.

Piralla, Antonio, Marco Zecca, Patrizia Comoli, Alessia Girello, Rita Maccario, and Fausto Baldanti. 2015. “Persistent Rhinovirus Infection in Pediatric Hematopoietic Stem Cell Transplant Recipients with Impaired Cellular Immunity.” *Journal of Clinical Virology* 67: 38–42. https://doi.org/https://doi.org/10.1016/j.jcv.2015.03.022.

Stott, E J, C Draper, P B Stones, and D A J Tyrrell. 1969. “Absence of Heterologous Antibody Responses in Human Volunteers after Rhinovirus Vaccination.” *Archiv Für Die Gesamte Virusforschung* 28 (1): 89–92. https://doi.org/10.1007/BF01250850.

Wisdom, A, E C McWilliam Leitch, E Gaunt, H Harvala, and P Simmonds. 2009. “Screening Respiratory Samples for Detection of Human Rhinoviruses (HRVs) and Enteroviruses: Comprehensive VP4-VP2 Typing Reveals High Incidence and Genetic Diversity of HRV Species C.” *Journal of Clinical Microbiology* 47 (12): 3958 LP-3967. https://doi.org/10.1128/JCM.00993-09.
